# Recommendations Regarding Skin Cancer Prevention from the Community Preventive Services Task Force

**Published:** 2014-05-23

The Community Preventive Services Task Force has posted new information online regarding prevention of skin cancer. Recommendations are offered regarding interventions for child care centers, primary and middle schools, high schools and colleges, outdoor occupational or recreational and tourism settings, and mass media and multicomponent communitywide campaigns. Summaries and links to the individual recommendations are available at http://www.thecommunityguide.org/cancer/skin/education-policy/index.html and http://www.thecommunityguide.org/cancer/skin/community-wide/index.html.

Established in 1996 by the U.S. Department of Health and Human Services, the task force is an independent, nonfederal, uncompensated panel of public health and prevention experts whose members are appointed by the Director of CDC. The task force provides information for a wide range of decision makers on programs, services, and policies aimed at improving population health. Although CDC provides administrative, research, and technical support for the task force, the recommendations developed are those of the task force and do not undergo review or approval by CDC.

